# Relationships between Traumatic Symptoms and Environmental Damage Conditions among Children 8 Months after the 2011 Japan Earthquake and Tsunami

**DOI:** 10.1371/journal.pone.0050721

**Published:** 2012-11-29

**Authors:** Masahide Usami, Yoshitaka Iwadare, Masaki Kodaira, Kyota Watanabe, Momoko Aoki, Chiaki Katsumi, Kumi Matsuda, Kazunori Makino, Sonoko Iijima, Maiko Harada, Hiromi Tanaka, Yoshinori Sasaki, Tetsuya Tanaka, Hirokage Ushijima, Kazuhiko Saito

**Affiliations:** 1 Department of Child and Adolescent Psychiatry, National Center for Global Health and Medicine, Kohnodai Hospital, Ichikawa, Japan; 2 Department of Psychiatry, Kitasato University, Sagamihara, Japan; 3 Kumamoto University Graduate School of Medicine, Department of Psychiatry, Kumamoto, Japan; Sapienza University of Rome, Italy

## Abstract

**Background:**

To evaluate relationships between traumatic symptoms and environmental damage conditions among children who survived the 2011 Great East Japan Earthquake and Tsunami.

**Methods:**

The subjects were 12,524 children in kindergartens, elementary schools, and junior high schools in Ishinomaki City, Miyagi Prefecture, Japan. The Post Traumatic Stress Symptoms for Children 15 items (PTSSC-15), a self-completion questionnaire on traumatic symptoms, was distributed to the children and a questionnaire regarding environmental damage conditions affecting the children was distributed to their teachers. Of 12,524 questionnaires distributed, an effective response was obtained from 11,692 (93.3%).

**Results:**

The PTSSC-15 score was significantly higher in females than in males among 4^th^ to 6^th^ grade students in elementary schools and among junior high school students. In terms of traumatic symptoms and environmental damage conditions, with the exception of kindergartners, children who had their houses damaged or experienced separation from family members had a significantly higher PTSSC-15 score than children who did not experience environmental damage. Except for kindergartners and 4^th^- to 6^th^-grade elementary school students, children who experienced evacuation had a significantly higher PTSSC-15 score.

**Conclusions:**

This study demonstrated relationships between traumatic symptoms and environmental damage conditions in children who had suffered from the disaster. Factors examined in studying the relationship between environmental damage conditions and traumatic symptoms were gender, age, house damage, evacuation experience, and bereavement experience. It was critical not only to examine the traumatic symptoms of the children but also to collect accurate information about environmental damage conditions.

## Introduction

On March 11th, 2011, Japan was struck by a huge earthquake and tsunami. The tsunami caused tremendous damage and victimized a number of children[1]–[4]. To date, there have been a number of studies of children who have survived disasters[5]–[10]. Studies of psychiatric problems in children who experienced the South East Asian tsunami in 2004 were reviewed [11]–[19].

After any disaster, posttraumatic stress disorder (PTSD) is the psychiatric diagnosis that should be considered most carefully [7], [18]–[21]. However, traumatic symptoms tend to heal spontaneously over time, and so the morbidity of PTSD is dependent on time, the subjects, and the methods used in the survey [6], [8], [14], [18], [19], [22]–[25].

We collected information on the traumatic symptoms and environmental damage conditions experienced by children who lived through the earthquake and tsunami eight months after the disaster (November 2011). The information included: gender, age, and environmental damage conditions. This was gathered in the hope of enabling the investigation of relationships between environmental damage conditions and traumatic symptoms eight monthsafter exposure [8], [20], [26].

The aim of this study was to evaluate relationships between traumatic symptoms and environmental damage conditions in children eight months after the earthquake and tsunami.

**Table 1:** Characteristics of children who experienced the 2011 Japan Earthquake and Tsunami.

Items			N = 11639	
Gender	Male		5939	(51.0%)
	Female		5700	(49.0%)
Age at the time of the disaster (y) (Mean)			10.9	(SD = 2.7)
PTSSC-15 score (Mean)			20.5	(SD = 14.5)
PTSSC-15 score at the cut-off value or higher (23 points)			4956	(42.6%)
House damage	No		6986	(60.0%)
	Yes	Total collapse	2243	(19.3%)
		Half collapse	2354	(20.2%)
		Total	4597	(39.5%)
	Unknown		56	(0.5%)
Evacuation experience	No		8228	(70.7%)
	Yes	Currently living in evacuation center	90	(0.8%)
		Used to live in evacuation center	2845	(24.4%)
		Living in temporary housing	976	(8.4%)
		Used to live in temporary housing	51	(0.4%)
		Evacuation experience at least once	3248	(27.9%)
	Unknown		163	(1.4%)
Bereavement experience	No		9241	(79.4%)
	Yes	Father	71	(0.6%)
		Mother	66	(0.6%)
		Brothers and sisters	44	(0.4%)
		Grandfather and grandmother	355	(3.1%)
		Classmates	1498	(12.9%)
		Teacher in charge	32	(0.3%)
		Others	270	(2.3%)
		At least one bereavement experience	2103	(18.1%)
	Unknown		295	(2.5%)

M, mean; SD, standard deviation; N, number of cases.

## Materials and Methods

### Study Design and Setting

This study involved the observation of relationships between traumatic symptoms and environmental damage conditions among children after the 2011 Japanese earthquake and tsunami. Ishinomaki City is the second largest city (population, 162,822) in Miyagi Prefecture, Japan. As of February 15, 2012, the death toll in Ishinomaki City was 3,182 and 557 people were missing. The total number of collapsed houses and buildings, including half-collapsed houses, was 33,378 and 7,298 temporary houses had been constructed.

**Table 2:** Average PTSSC-15 score (grade & gender).

	Gender							
	Male			Female				
	M	IR	N	M	IR	N	Effect size	P-value
Kindergartners	14.0	3.0–25.5	119	15.0	3.0–25.5	127	0.03	ns
1st – 3rd gradeelementary school students	16.0	6.0–28.0	1866	17.0	7.0–28.0	1736	0.03	ns
4th – 6th gradeelementary school students	17.0	7.0–29.0	1975	20.0	9.0–32.0	1973	0.08	<0.0001
Junior high school students	20.0	9.0–32.0	1979	26.0	14.0–37.0	1864	0.15	<0.0001

M, median; IR, interquartile range; N, number of cases.

### Recruitment and Participants

This survey was conducted as part of the school education program under the initiative of the Board of Education in Ishinomaki City. Surveys were distributed to all children who attended five kindergartens, 43 elementary schools, and 21 junior high schools in Ishinomaki City, Miyagi Prefecture. The survey was carried out in November 2011 (eight months after the earthquake disaster) after temporary houses had been provided for all evacuees in need in Ishinomaki City and after all evacuation centers had been closed.

**Table 3:** Average PTSSC-15 score (grade & gender & house damage).

Grade Group	Gender	House damage								F	p value
		Absent			Present						
		M	SD	N	M		SD	N			
Kindergartners									Gender×House damage	0.32	ns
	Male	14.94	12.80	95	16.50		11.63	24	House damage	1.67	ns
	Female	14.90	12.10	109	18.89		15.26	18	Gender	0.30	ns
1st – 3rd gradeelementaryschool students									Gender×House damage	0.26	ns
	Male	16.03	12.10	1130	19.49		13.39	712	House damage	54.63	<0.0001
	Female	17.06	12.44	1053	20.07		13.28	658	Gender	3.38	ns
4th – 6th gradeelementaryschool students									Gender×House damage	0.01	ns
	Male	18.90	14.00	1128	20.11		14.73	846	House damage	6.13	0.0134
	Female	21.12	14.38	1169	22.23		15.15	800	Gender	21.44	<0.0001
Junior highschool students									Gender×House damage	0.96	ns
	Male	20.67	14.71	1194		22.37	14.94	783	House damage	5.86	0.0155
	Female	25.48	15.40	1108	26.20		15.79	756	Gender	74.70	<0.0001

M, mean; SD, standard deviation; N, number of cases.

First, the survey method was explained to the principals of all of the schools by the Education Committee of Ishinomaki City. Then teachers distributed a letter explaining the survey, which had been constructed by the Education Committee, to all children and their parents. The letter clearly stated that the students’ filling out the questionnaire would be considered for both the parents and the students as having given consent to the survey. The letter also specified that the survey results would be used to provide children with psychological care to facilitate their education at school and that the results would be published as a medical paper. Informed consent was obtained when the students filled out the questionnaire. This consent procedure was approved by the ethical committee of the National Center for Global Health and Medicine.

**Table 4:** Average PTSSC-15 score (grade & gender & evacuation experience).

Grade Group	Gender	Evacuation experience							F	p value
		Absent			Present					
		M	SD	N	M	SD	N			
Kindergartners								Gender×Evacuation	0.1643	ns
	Male	14.73	12.58	105	19.14	11.99	14	Evacuationexperience	1.791	ns
	Female	15.21	12.11	112	17.57	16.68	14	Gender	0.04644	ns
1st – 3rd gradeelementary school students								Gender×Evacuation	0.07718	ns
	Male	16.45	12.30	1401	20.25	13.58	436	Evacuationexperience	52.75	<0.0001
	Female	17.39	12.50	1311	20.91	13.68	395	Gender	2.52	ns
4th – 6th gradeelementary school students								Gender×Evacuation	0.0115	ns
	Male	19.36	14.16	1375	20.30	14.60	557	Evacuationexperience	3.763	0.0525
	Female	21.35	14.66	1357	22.40	14.94	577	Gender	15.9	<0.0001
Junior highschool students								Gender×Evacuation	0.2867	ns
	Male	20.91	14.91	1342	22.25	14.63	624	Evacuationexperience	9.597	0.0020
	Female	25.18	15.38	1225	27.08	15.86	631	Gender	75.7	<0.0001

M, mean; SD, standard deviation; N, number of cases.

The Posttraumatic Stress Symptoms for Children 15 items (PTSSC-15), a self-completion questionnaire on traumatic symptoms, was distributed to 12,524 children registered at municipal schools in Ishinomaki City, and a questionnaire on the environmental damage experienced by the children was distributed to their teachers. Parents of kindergartners and 1^st^- to 3^rd^-grade elementary school students were asked to fill out the questionnaire while talking with their children. Informed consent for participation in the survey was obtained at the time that the completed questionnaires were received from the children.

**Table 5:** Average PTSSC-15 score (grade & gender & bereavement experience).

Grade Group	Gender	Bereavement experience							F	p value
		Absent			Present					
		M	SD	N	M	SD	N			
Kindergartners								Gender×bereavementexperience	0.11	ns
	Male	15.02	11.98	94	16.12	14.64	25	Bereavementexperience	0.69	ns
	Female	15.26	12.57	110	17.81	12.97	16	Gender	0.19	ns
1st – 3rd gradeelementaryschool students								Gender×bereavementexperience	0.56	ns
	Male	16.77	12.56	1515	20.28	13.21	299	Bereavementexperience	27.35	<0.0001
	Female	17.80	12.67	1418	20.43	13.60	265	Gender	1.01	ns
4th – 6th gradeelementaryschool students								Gender×bereavementexperience	0.01	ns
	Male	19.08	14.37	1512	20.77	14.18	419	Bereavementexperience	8.91	0.0028
	Female	21.26	14.59	1430	22.87	14.94	483	Gender	15.00	<0.0001
Junior highschool students								Gender×bereavementexperience	1.75	ns
	Male	20.94	14.86	1628	23.57	14.39	303	Bereavementexperience	27.21	<0.0001
	Female	25.16	15.31	1534	29.57	16.21	293	Gender	57.33	<0.0001

M, mean; SD, standard deviation; N, number of cases.

**Table 6:** Experience of separation from various individuals and average PTSSC-15 score.

	Male			Female		
	M	SD	N	M	SD	N
Father	20.38	14.52	39	24.75	14.45	32
Mother	23.66	13.30	32	34.06	14.26	34
Brother/Sister	22.35	16.68	20	29.04	11.86	24
Grandparent	22.24	14.09	158	22.41	14.67	197
Classmate	21.57	14.21	766	23.67	15.25	732
Teacher	22.05	12.33	19	30.23	11.62	13
Other	20.56	14.43	127	23.20	16.30	143

M, mean; SD, standard deviation; N, number of cases.

Answers were returned from 12,346 (98.6%) of the 12,524 children to whom questionnaires were distributed. Of the 12,346 children, effective response was obtained from 11,692 (93.3%). Answers for environmental damage with regard to all 12,524 children were returned from teachers.

**Table 7:** Average PTSSC-15 score (number of disaster experiences and gender).

Number of disaster experiences	Male			Female				F	P Value
	M	SD	N	M	SD	N			
0	18.24	13.82	2985	20.51	14.41	2829			
1	19.75	14.03	1355	22.09	14.61	1330	Gender×disaster experience	0.82	ns
2	20.26	14.27	1148	23.44	14.94	1066	Disaster experience	34.98	<0.0001
3	23.16	14.50	443	24.90	16.04	460	Gender	52.40	<0.0001

M, mean; SD, standard deviation; N, number of cases.

### Measures

A paper-based survey was conducted, asking questions regarding traumatic symptoms using a self-report form.

The self-report form consisted of the PTSSC-15 and a daily life questionnaire developed by the authors. The teacher-report form consisted of a disaster situation questionnaire for each student developed by the authors.

### PTSSC-15

The PTSSC-15 is a self-completion questionnaire on the stress reactions in children after disasters. Posttraumatic Stress Symptom 10(PTSS10) [27] had fewer questions and was used as a screening test after the Hanshin Great Earthquake and is familiar in Japan [21]. In 105 Norwegian children (6–17 years old) devastated by the 2004 South East Asia Tsunami, PTSS10 was administered 10 and 30 months after the disaster [28]. Five questions that were considered to be important psychosomatic characteristics after disasters (flashback, appetite loss, somatic reaction such as headache and abdominal pain, attention deficit, and anxiety) were added to the PTSS10, and the PTSSC-15 consisting of 15 questions was constructed in Japan [29].

Each question is scored in six levels: 0 = completely disagree, 1 = mostly disagree, 2 = partially disagree, 3 = partially agree, 4 = mostly agree, and 5 = completely agree. Higher scores indicate more severe traumatic symptoms and depressive symptoms. Kishi et al [29] demonstrated the reliability and validity of the PTSSC-15 for Japanese children and adolescents. The cut-off value for the presence of PTSD or being at high risk for PTSD is 23 points in Japan [29].

### Environmental Damage Conditions

The authors and the educational committee in Ishinomaki City developed the questionnaire regarding environmental damage conditions experienced by the subject children. The form was designed to be completed by teachers. It asked about the conditions of disaster damage, bereavement experience, and life in evacuation centers. With regard to the environmental damage conditions of the children’s houses, one of the following three answers was selected: “no damage”, “total collapse by the earthquake or tsunami (incapable of living in the house)”, “half collapse by the earthquake or tsunami (necessary to repair the house in order to live in it)”.

Regarding the living conditions in evacuation centers, multiple-choice questions and answers were selected from the following options: “no experience”, “currently living in the evacuation center”, “used to live in the evacuation center”, “living in a temporary house”, and “used to live in a temporary house”.

As to the bereavement experience (including the experience of unexplained disappearance due to the earthquake), multiple answers were allowed from the following eight responses: “no experience”, “father”, “mother”, “brothers and sisters”, “grandfather and grandmother”, “kindergarten and school classmates at the time of the earthquake”, “teacher in charge of the class at the time of the earthquake”, and “others”.

## Statistical Analysis

### PTSSC-15, School Grades, and Gender

Children were divided into four grade groups: kindergartners, lower-grade (1^st^ to 3^rd^ grade) elementary school students, higher-grade (4^th^ to 6^th^ grade) elementary school students, and junior high school students (7^th^ to 9^th^ grade). In each grade group and gender, the median PTSSC-15 score, and interquartile range were determined. The PTSSC-15 score was statistically compared between males and females by the Mann-Whitney U test for each grade group. Effect sizes were calculated based on the Mann-Whitney statistics.

### PTSSC-15 and Environmental Damage Conditions

With regard to environmental damage conditions, the house damage, evacuation conditions, and bereavement experience were examined. Then the average PTSSC-15 score in each grade group and gender was calculated. As to the bereavement experience, the average PTSSC-15 score was calculated separately in the subjects who experienced bereavement. Children were categorized by house damage, evacuation conditions, and bereavement experience, and the difference in the average PTSSC-15 score between the groups was statistically analyzed by two-factor analysis of variance in each grade group and gender. In addition, the number of disaster experiences (house damage, evacuation conditions, and bereavement experience) of the children was examined and compared with the average PTSSC-15 score in each gender.

In all tests, a significance level of 0.05 was used in two-sided tests. Analyses were performed using PASW 18.0.

## Results

### Descriptive Information

The participants included 11,692 children [5959 males, 5733 females] who were exposed to the 2011 Japanese earthquake and tsunami. Table 1 shows the gender, score of PTSSC-15, age, and environmental damage conditions (house damage, evacuation conditions, and bereavement experience) in 11,692 children. When teachers had no information about house damage, evacuation conditions, and bereavement experience, the answer was defined as “unknown”.

### Traumatic Symptoms, Gender, and Age

Table 2 shows the average PTSSC-15 score in each grade group and gender. The PTSSC-15 score was significantly higher (p<0.001) in girls than in boys in the higher grade of elementary schools and junior high schools. These effect sizes were under 0.20.

### Traumatic Symptoms and Environmental Damage Conditions

For comparison of traumatic symptoms and environmental damage conditions, the variables of house damage, evacuation experience, and bereavement experience were examined individually. The average PTSSC-15 score and having experienced house damage were compared in each grade group and gender (Table 3). Children in all age groups, except kindergartners, who had house damage had a significantly higher PTSSC-15 score than children without it (F(1,3567) = 54.63, p<0.001; F(1,3951) = 6.126, p<0.0013; F(1,3864) = 5.86, p<0.0016).

With regard to the evacuation experience, the relationship between the average PTSSC-15 score and evacuation experience is shown in Table 4 in each grade group and gender. Except kindergartners and higher-grade elementary school students, children who had evacuation experience showed a significantly higher PTSSC-15 score than children without it (F(1,3539) = 52.75, P<0.0001; F(1,3818) = 9.60,P<0.002).

Regarding the bereavement experience, the average PTSSC-15 score and bereavement experience are shown in Table 5 in each grade group and gender. Except for kindergartners, children who had experienced bereavement had a significantly higher PTSSC-15 score than children who had not (F(1,3493) = 27.35, p<0.0001; F(1,3840) = 8.91, p<0.0028; F(1,3754) = 27.21, p<0.0001). In addition, Table 6 shows the average PTSSC-15 score in children who had experienced the loss of various relationships.

Table 7 shows the number of disaster experiences and the average PTSSC-15 score for each gender (F(3,11608) = 34.98, p<0.0001). There was a significant difference in the PTSSC-15 score with and without disaster experience and between males and females (F(3,11608) = 52.40, p = <0.0001).

## Discussion

This study showed relationships between traumatic symptoms and environmental damage conditions in children who had experienced damage and loss eight months after a severe natural disaster.

The number of children with a PTSSC-15 score higher than the cut-off value was 42.6%. Using only a self-completion questionnaire as a screening tool for PTSD after a huge disaster may result in an inflated number of children who appear to be at high risk for PTSD. Collecting information about the environmental damage experienced by the children and correlating that with their scores on the questionnaire may result in a more accurate prediction of which children are at high risk for PTSD.

Differences in traumatic symptoms due to gender and age were recognized in this study. According to the effect size, the difference due to gender is negligible, but difference due to age is large. The responders in the case of kindergartners and lower-grade elementary school students were the parents, and there is a possibility that not only the status of the children but also the psychological anxiety and status of their parents affected the responses. Therefore, it is necessary to comprehensively judge the status of children by considering not only their psychological status but also the psychological conditions of their parents.

We found that there were more severe traumatic symptoms in children who had experienced house damage and evacuation. House damage and evacuation experience as a result of the tsunami and earthquake may have directly influenced the psychological condition of the children in this study.

Furthermore, we found that bereavement experience was closely related to traumatic symptoms of the children. In particular, the results of this study suggest that children who lost their mother had traumatic symptoms most frequently among children with bereavement experience, and disappearance of the mother may have a great influence on the emotional growth of the child.

In studies on children after disaster damage, in addition to evaluating their direct experience of the earthquake and tsunami with regard to its size and magnitude, the impact of house damage, evacuation conditions, bereavement experience, and changes in family composition by the earthquake disaster, should be carefully investigated.

### Limitations

This study was a survey with a self-completion questionnaire carried out in only one district in Japan and it is impossible to calculate the morbidity of PTSD in children after the 2011 Japanese earthquake and tsunami based on the results of the survey. Therefore, this study is insufficient as an epidemiological survey for psychiatric diagnosis. Examinations by child psychiatrists using operational diagnostic criteria and structured interviews are necessary for accurate psychiatric diagnosis. Moreover, the results of this study on children in Ishinomaki City do not reflect all characteristics of children who experienced the 2011 Japanese earthquake and tsunami.

### Conclusion

This study elucidated relationships between traumatic symptoms and environmental damage conditions in children who survived the earthquake and tsunami. It is important not only to evaluate the traumatic symptoms with a self-completion questionnaire but also to confirmspecific information about the extent of environmental damage conditions that the children experienced after the disaster.
